# Categorisation of built environment characteristics: the trouble with tertiles

**DOI:** 10.1186/s12966-015-0181-9

**Published:** 2015-02-15

**Authors:** Karen E Lamb, Simon R White

**Affiliations:** Centre for Physical Activity and Nutrition Research, Deakin University, Burwood, VIC 3125 Australia; Medical Research Council Biostatistics Unit, Cambridge Institute of Public Health, Cambridge, CB2 0SR UK

**Keywords:** Percentile categorisation, Exposure assessment, Built environment, Neighbourhood, Statistical analysis

## Abstract

**Background:**

In the analysis of the effect of built environment features on health, it is common for researchers to categorise built environment exposure variables based on arbitrary percentile cut-points, such as median or tertile splits. This arbitrary categorisation leads to a loss of information and a lack of comparability between studies since the choice of cut-point is based on the sample distribution.

**Discussion:**

In this paper, we highlight the various drawbacks of adopting percentile categorisation of exposure variables. Using data from the SocioEconomic Status and Activity in Women (SESAW) study from Melbourne, Australia, we highlight alternative approaches which may be used instead of percentile categorisation in order to assess built environment effects on health. We discuss these approaches using an example which examines the association between the number of accessible supermarkets and body mass index.

**Summary:**

We show that alternative approaches to percentile categorisation, such as transformations of the exposure variable or factorial polynomials, can be implemented easily using standard statistical software packages. These procedures utilise all of the available information available in the data, avoiding a loss of power as experienced when categorisation is adopted.We argue that researchers should retain all available information by using the continuous exposure, adopting transformations where necessary.

## Background

Interest in the effect of the built environment on obesity and related behaviours has grown over the last fifteen years [1], with geographic information system software allowing objective measures of neighbourhood resources to be linked to health outcomes. Much research has considered benefits of access to presumed “healthy” resources, such as supermarkets [2,3] which provide nutritious foods, and sports centres [4] where physical activity is undertaken, and “unhealthy” resources such as fast-food outlets [2,3] which sell high calorie content products. Built environment attributes (e.g., street connectivity or land use) which may promote healthy behaviours such as walking have also been examined [5].

Obtaining comparable estimates of the effect of environmental attributes across studies is challenging. While evidence on *perceived* environmental features on obesity related outcomes such as physical activity has been pooled [6], we are unaware of any meta-analyses of the effect of objective built environment features. Papas et al. [7] highlighted a number of methodological limitations which prevent studies from being pooled, including differences in the conceptualisation of measures of the built environment, whether it be the type of feature under consideration (e.g., food outlets, walkability), the scale at which measures are considered (e.g., 1 km buffer, census tract), or the choice of measurement (e.g., distance to resource or density of resources). While these all provide great challenges, a further issue when comparing studies is the seemingly arbitrary categorisation of variables. In this article we highlight, with the aid of an illustrative example from the Socio-Economic Status and Activity in Women (SESAW) study in Melbourne, Australia [8], why categorisation should be avoided and discuss alternative analytical approaches.

## Discussion

### Categorisation of built environment characteristics

While the title of our article draws attention to the use of tertiles, somewhat akin to the “disappointing dichotomies” raised in the clinical context [9], we could equally have entitled this piece “quarrels with quartiles” or “quandaries with quintiles”; all of these approaches of exposure categorisation have been adopted in analyses of built environment effects on health. Recent literature provides examples examining binary splits (at the median or upper quartile) [10-12], tertiles [13-15], quartiles [16,17], quintiles [18-20], or some other data-dependent categories [21,22].

A recent British Medical Journal article [23], which examined access to takeaway food outlets in different exposure settings, highlights one of our key concerns with categorisation: difficulties in drawing comparisons. Burgoine et al. used quartiles of access to outlets across three different exposures resulting in ‘low exposure’ being zero outlets around the home, less than three around work, and less than two in the commuting environment; meaning that the definition of ‘low exposure’ differed by category, making direct comparisons between the three exposure environments difficult.

In many of these studies it appeared that the researchers had the continuous data available but chose to categorise them. Typically no clear rationale for the categorisation was provided [13,15,17,19,20], or it was used for “ease of interpretation” [11], or to allow comparisons of approach and results to other studies [12]. In one case, after finding no departure from linearity, quartiles were used to test for threshold effects but no justification as to why quartiles were adopted for this purpose was provided [16]. Elsewhere, categorisation was used to examine linearity in associations [18], while another study used dichotomisation when a bivariate distribution was apparent and a median split where this was not [10].

We should acknowledge at this point that the authors are not blameless, having used categories of exposure in the past. However, given the lack of consensus on defining low, medium and high exposure, we thought it prudent to highlight the drawbacks of categorising continuous exposure variables and our thoughts on future analytic directions in this field.

### Costs of categorisation

While the costs of categorisation are frequently raised in clinical literature [24-29] and dichotomisation has been discussed in psychology literature [30], these issues have not been emphasised in social epidemiology, in particular when examining effects of the built environment on health where percentile categorisation commonly occurs.

As discussed in other critiques of categorisation, often authors use dichotomies due to arguments of simplicity, avoiding assumptions about the nature of the relationship between the predictor and outcome variable and to deal with skew or outliers in the exposure distribution [27-29] and this argument is extended to the use of other levels of categorisation [25]. However, there are numerous reasons why categorisation should be avoided, in particular categorisation based on cut-points of the predictor distribution such as tertiles, quartiles and quintiles.

Firstly, categorisation leads to a loss of power when examining predictor-outcome associations. Although this is greatest when considering smaller numbers of categories (for example, dichotomising a normally distributed predictor variable at the median results in an effective loss of approximately a third of the data! [28]), a loss of power occurs whenever categorisation is adopted. Given that studies of the built environment and health frequently involve numerous built environment exposure variables, researchers should aim to avoid the loss of power attributable to the arbitrary categorisation across these variables. Considering the extreme case of dichotomisation of multiple predictors, Royston et al. [28] highlighted the difficulty in determining what will occur when more than one predictor variable is dichotomised, noting that this could lead to spurious associations or interactions between predictor variables and stressing that these problems could be more severe if the cut-points are chosen according to median splits or some other data-dependent approach rather than chosen *a priori* based on some meaningful threshold.

This data-dependent approach to categorisation leads to our second concern; namely, that the choice of cut-points is biased. Without any prior rationale for the choice of categorisation, the cynical researcher may speculate that the authors simply chose to present the categorisation which led to the finding of a statistically significant result. In truth, this type of approach is problematic due to the issue of multiple testing which could actually render the result not significant at the pre-specified p-value threshold. Furthermore, adopting this type of analytical approach is unlikely to find a threshold which is meaningful beyond the sample for which it was derived. The approach of testing multiple cut-points to determine which produces the most significant result (i.e., smallest p-value) was advocated by Schulgen et al. [31]. However, the authors stress that adjustment for multiple testing should be adopted and that researchers should be transparent about this approach and the results obtained.

**Figure 1:**
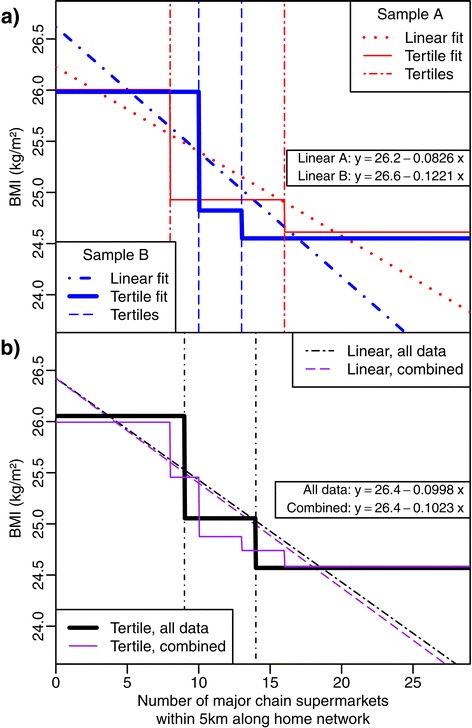
**Illustrative example of the ‘trouble with tertiles’ predicting BMI using the count of supermarkets.** We split the original SESAW dataset [3] (n = 1462) into two sub-samples, A and B, each with n = 500. **(a)** The sub-samples are analysed separately using a tertile approach and a linear model (with a single linear predictor and intercept, the linear fits are both significant and the coefficients are shown on the plot). **(b)** If we consider the two sub-samples as independent studies, it is then of interest to consider the combined estimate of the association between supermarket density and BMI. The combined sub-sample fits are obtained using standard meta-analysis methods (in essence, a weighted mean of the estimates accounting for sample size and standard errors); the combined fits are compared to the same analysis on the complete data. Of note, the combined tertile model no longer has three groups, there are now five groups, which complicates the interpretation. Conversely, the combined linear model retains the same interpretation.

Our third concern is that it is difficult to compare or replicate results between studies. Therefore it is difficult to pool evidence of the effect of a predictor on an outcome variable. We illustrate this issue in Figure 1 in which we consider the effect of the number of supermarkets within 5 km of home on body mass index (BMI) from the SESAW study. Two random sub-samples of SESAW data were considered and the tertiles compared. Note from Figure 1(a) how different the tertile ranges are. These sub-samples were recombined in Figure 1(b), pooling evidence as in a meta-analysis. Combined fits were computed and compared to the full data fit. Combining the continuous fits from the two sub-samples matches the continuous fit from the full sample very well, whereas using the sub-sample tertile fits it is impossible to recover the full data tertile fit.

Our final concern with data-dependent categories relates to the interpretation of the (often wide) intervals, specifically that any estimated effect applies constantly across the entire range of the category. Considering the SESAW example (Figure 1(a)), the effect on BMI (our outcome) is constant across the whole range of each tertile of supermarket access regardless of how wide the interval. So, for Sample A, the effect of having nine supermarkets within 5 km is assumed to be the same as having sixteen.

We have argued some of the pitfalls of categorisation. However, potential justifications of dichotomising data have been proposed. Although generally critical of the approach, MacCallum et al. [30] mention that in very rare circumstances dichotomisation may be justifiable. For example, if an analysis provided clear support for the existence of two taxonomies alongside a clear set point at which these two groups differ then the use of dichotomisation may be supportable. However, MacCallum et al. stress that this will still undoubtedly result in a loss of information. Furthermore, the use of arbitrary cut-points, such as the median split, will be most unlikely to identify the groupings.

### Alternative approaches

An often stated drawback of using the continuous predictor over categories is that this relies on normality assumptions and linear relationships between the predictor and the outcome [32]. However, Brenner and Blettner [26] discussed issues associated with categorisation in epidemiological studies showing that, even if model assumptions are violated, including the confounder as a linear variable typically controls confounding while, in contrast, residual confounding appears to be present if the variable is categorised, particularly when the number of categories is small (i.e., five or less). Thus the standard linear model with a continuous covariate is fairly robust (in a statistical sense). There are a number of approaches which can be used to investigate beyond simple linear relationships without resorting to data-dependent categories.

A first approach is to include transformations of variables as predictors in the model, for example the square of a variable. Using well-established model comparison techniques (e.g., Akaike Information Criteria (AIC)) we can test whether these additional transformed variables improve our fit to the data. Transformations can be particularly useful when dealing with highly skewed distributions or those featuring potential outlying or extreme observations as transformations can sometimes remove this skewness or draw extreme values closer to the sample distribution. An outlier is informally defined as a value (typically) greater than three standard deviations from the mean, a criteria based on the normal distribution. However, there is no requirement for independent variables to be normally distributed (for linear regression only the residuals need to be normally distributed). Thus it is always necessary to consider variables in context. For example, predictors which are counts can be represented as mixture distributions, where zeros are distinct from non-zeros, and these predictors can be assessed by considering a model which splits the count predictor into two variables (with a separate coefficient for both zero and non-zero counts). The fit of the model can then be assessed by performing a model comparison of a model which includes the split predictor and one which contains the predictor with no split.

To fully examine the effect of outliers on the results obtained, it is often worth conducting a sensitivity analysis in which outliers or extreme values are removed from the analysis in order to determine how much the results are influenced by these values. While at first glance the use of percentile categorisation, such as tertiles, appears to robustly account for outliers – as the cut-points are robust to outliers – this leads to problems interpreting the categorisation bands if the covariate is not defined over a closed interval (i.e., if the range of values is unbounded in either or both directions). For example, typically count covariates have a lower bound of zero but have no well-defined upper bound. A common mistake in reporting percentile categorisations is to state the bands as closed intervals when they are not. This leads to the problem of interpreting unbounded categories with outliers, as the outliers are forced to be equivalent to the other values in the same band. Thus, although the categories are robustly defined in the presence of outliers, within a percentile categorisation approach we lose the ability to investigate those same outliers. For example, if a single outlier has an exceptional response measurement then it might have an unusual residual. Under the percentile categorisation we would not be able to see anything unusual about the observation whereas by keeping the raw observation we would quickly detect the large residual was linked to the outlying observation.

Factorial polynomials [33] are a formalised approach to including a pre-specified set of transformations and performing model comparisons to select the best fitting model. Many statistical software packages (e.g., Stata, R) include automated factorial polynomial regression routines.

Beyond adding transformations of covariates we may truly consider a non-linear or non-parametric model. However, as with tertiles, we may complicate any subsequent attempts to combine separate studies. Two popular techniques are local regression and splines (e.g., cubic or B-splines). These techniques fit more complicated curves to the covariate data hoping to capture more complex relationships.

**Figure 2:**
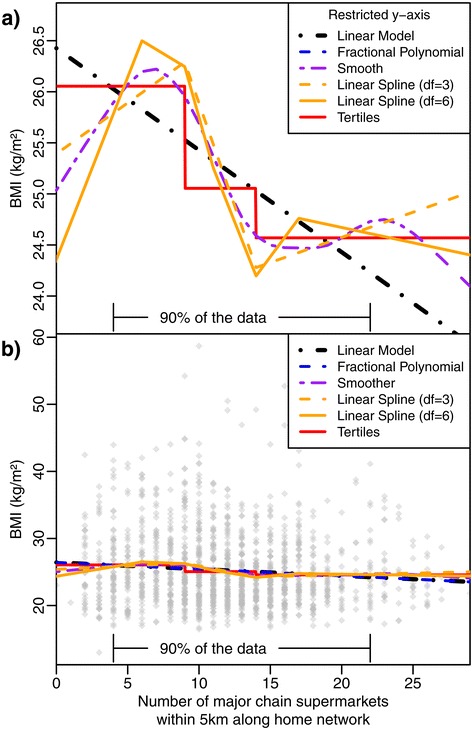
**Comparison of approaches for estimating non-linear relationships using the SESAW study [** 3 **].** **(a)** Comparison of a simple linear model, fractional polynomial (of which the best fitting was equivalent to the simple linear model), linear splines, tertiles and a non-parametric smoother (see Table 1 for the respective AICs to assess model comparison). **(b)** As in Figure 2(a) with an extension to the y-axis to show the complete range of BMI and the observed data plotted (n = 1462 points). We see visually the result of comparing the AICs in Table 1 that due to the large variance in BMI scores there is no evidence for anything more complicated than a simple linear model. Further, there is nothing statistically to choose between the linear and tertile fits. However, the linear model has the benefit of not being data-dependent.

Figure 2 shows the results from fitting alternative approaches for estimating non-linear relationships between supermarket access and BMI to the SESAW data: an automated fractional polynomial (which selected only the untransformed continuous covariate), a non-parametric smooth curve, and two linear splines with differing numbers of knots. The spline models fitted are for fixed-knot splines where, like the tertile model, the knot locations are typically data-dependent (although we could, and should, defined equi-spaced fixed-knot locations over a realistic range of the covariate). Conversely, it is possible to fit free-knot splines where both the number and location of the knots are inferred from the data. The methods for pooling multiple spline models are more complex than simple linear models (involving evidence-synthesis [34] or multivariate meta-analysis [35]), so it must always be checked that their added complexity is warranted (i.e., model comparisons such as AIC). All fits shown in Figure 2 exhibit a similar pattern. Comparison of these models using AIC or Cox tests for non-nested models resulted in weak evidence that more complex non-linear models are required. Thus, the simple linear model provided a sufficient fit in our example, as shown in Figure 2(b) where the variation in BMI is clearly far greater than the model discrepancies. Modelling results are presented in Table 1 in which it can be seen that there is a statistically significant (though perhaps not clinically significant in terms of the effect on BMI) association between the number of supermarkets within 5 km and BMI using the predictor as a continuous variable or in tertiles. However, from Table 1, it is not clear when using tertiles if the relationship is linear or not and we cannot be sure how robust and reproducible the ‘low’, ‘medium’ and ‘high’ bands are. The benefit of not adopting a percentile categorisation approach by using methods for fitting non-linear relationships and model comparison approaches using AIC was that we were able to conclude that a linear association provided the best fit to the data and have this well-defined across the full range of data.

**Table 1:** **Comparison of modelling approaches for predicting BMI from the count of supermarkets within 5 km**

**Model**	**Predictor**	**Coefficient**	**(S.E.)** ^**(a)**^	**p-value**	**AIC** ^**(f)**^
Linear Model	Intercept	26.43	(0.35)	<0.001	9139.73
	Count of supermarkets	−0.10	(0.03)	<0.001	
Fractional	Intercept	26.53	(0.37)	<0.001	9139.73
Polynomial^(b)^	(Count of supermarkets + 1)/10	−1.00	(0.27)	<0.001	
Spline (2 knots)^(c)^	Intercept	26.53	(0.56)	<0.001	9141.66
	1st segment, 0—11 supermarkets	−1.25	(0.69)	0.068	
	2nd segment, 11—15 supermarkets	−4.80	(1.56)	0.002	
Spline (3 knots)^(d)^	Intercept	25.39	(0.68)	<0.001	9132.98
	1st segment, 0—9 supermarkets	0.90	(0.86)	0.29	
	2nd segment, 9—15 supermarkets	−1.11	(0.71)	0.12	
	3rd segment, 15—50 supermarkets	0.66	(0.66)	0.78	
Tertiles^(e)^	0—9 supermarkets (baseline)	26.05	(0.25)	<0.001	9137.67
	10—14 supermarkets	−1.00	(0.34)	<0.001	
	15— supermarkets	−1.49	(0.36)	<0.001	

^(a)^S.E. = standard error.

^(b)^The fractional polynomial with intercept and covariate was found to be the best fitting from among the pre-defined set of fractional polynomials (selection is based on the AIC and is automatically carried out by the statistical algorithm). Since the logarithm is one of the possible transformations, it is not allowed to have zero values, hence the addition of a 1 to the number of supermarkets in this model.

^(c)^Fixed 2-knot spline not shown on Figure 2.

^(d)^Default knots for the spline function are placed at the equivalent quantiles. Hence the knot locations coincide with the tertile boundaries. With splines, it is possible to estimate the knot locations as part of the inference or to use pre-specified knot locations. The spline was anchored to be within the range of 0 and 50 for this example.

^(e)^Note that the third category is unbounded. This highlights the issue of how outliers are included in the analysis and the issue of how to interpret a ‘high’ density of supermarkets, we can define high as 15, 20, 25, 30, etc. (the actual range of the data is 0—29). For closed intervals like these, the representative value can be thought of as the interval mid-point. However, taking the mid-point assumes values are uniformly distributed within the interval. For the lower band this is not true (low counts have a mean of 6.2 and median of 6 compared to the mid-point of 4.5). High counts have a mean of 18.3 and a median of 17 with an undefined mid-point due to the unspecified upper bound of percentile categorisation.

^(f)^We performed a Cox test for non-nested models to compare the model fits and found no significant difference in AIC between the linear and tertile model fits. The 3-knot spline has a smaller, therefore better, AIC but none of the coefficients are significant which perhaps indicates over-fitting. The 2-knot spline, with the same number of parameters as the tertile model, is not statistically different from the linear model.

**Figure 3:**
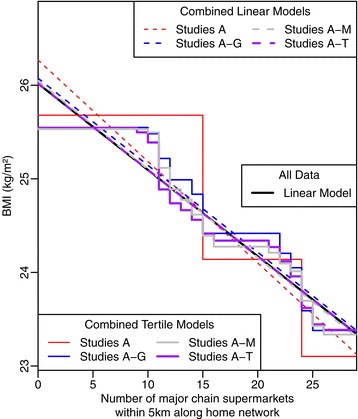
**Illustration that within a meta-analysis the tertile approach will tend to a linear model.** The SESAW data [3] were split into 20 sub-samples (A-T), each with n = 75. This plot shows four meta-analyses which combine an increasing number of the sub-samples (A, A-G, A-M, and A-T). The linear model approach is consistent and approaches the equivalent analysis using the full data. Conversely, the tertile approach becomes increasingly bumpy, as each sub-sample has data-dependent tertile cut-points. In the limit, as illustrated, the tertile combined analysis will tend towards the linear model approach.

Using our illustrative example, in Figure 3 we highlight the problems when pooling results from studies which use tertiles of the exposure variable, comparing results against pooling studies which used the supermarket access predictor as a continuous variable. This figure shows that the linear model approach is consistent and the meta-analysis of the sub-samples approaches the equivalent analysis when fitting the model using the full data, while the meta-analysis of the tertile exposure studies becomes increasingly bumpy since each sub-sample has data-dependent tertile cut-points.

The use of the continuous exposure measure allows us to assess the association between a one unit increase (i.e., an additional supermarket in this example) on BMI which will enable us to gain better understanding of whether or not increasing the number of supermarkets accessible from home is related to reductions in BMI. Of course, it must be borne in mind that while we advocate the use of continuous exposure measures in order to compare results across studies, it is important to think about the context of studies considering built environment features on health. Using our example of supermarket access on BMI, it would appear feasible to draw comparisons between the presence of an additional supermarket within the home neighbourhood in different studies regardless of the setting of the study. However, the definition of neighbourhood, and what is a reasonable distance to travel from home, may differ depending on context. That is, an individual’s perception of neighbourhood may differ dependent on the environmental context, with a 400 m distance in Melbourne, for example, perceived of differently to a 400 m distance in Hong Kong. While considering context is important, we feel that the use of continuous exposure measures will help elucidate these differences, highlighting where built environment features are important in different contexts.

Although we have drawn on examples from built environment effects on health in this article due to our knowledge of the use of this approach in this field, it is worth highlighting that this issue is not restricted to this area of research in social epidemiology. Percentile categorisation occurs frequently when dealing with other exposures such as dietary measures, physical activity exposures and many other predictors. Thus, the approaches discussed in this article are of relevance to other researchers within social epidemiology and behavioural research.

### Summary

Categorisation of exposure variables leads to a variety of problems, namely a loss of power, a potential for bias, a lack of replicability between studies, and an assumption that the estimated effect applies constantly across the entire range of the category. However, although there are many issues associated with percentile categorisation, their use appears frequently in research on effects of the built environment on health. While categorisation may seem appealing in the face of skewed distributions and non-linear relationships between exposure and outcome, we have shown that alternative analysis techniques are available which can be implemented to deal with such data. We strongly advocate that researchers in this field utilise all of the data available to them by using continuous exposure variables. This will greatly advance our ability to draw comparisons between studies of built environment effects on health.

## Abbreviations

SESAW: SocioEconomic Status and Activity in Women
BMI: Body mass index
AIC: Akaike information criteria
